# Supplementary material for: Structural basis for DNA proofreading
Source: Nat Commun. 2023 Dec 27;14:8501. doi: 10.1038/s41467-023-44198-8 (PMC10752894; doi:10.1038/s41467-023-44198-8)
Supplement: Supplementary file 1 — Supplemental Information [file 41467_2023_44198_MOESM1_ESM.pdf]

Supplementary Information for:
Structural Basis for DNA Proofreading

Gina Buchel*, Ashok R.Nayak*, Karl Herbine, Azadeh Sarfalah, Viktoriia Sokolova, Angelica Zamudio-Ochoa, and Dmitry Temiakov

Department of Biochemistry and Molecular Biology,
Thomas Jefferson University; 1020 Locust St, Philadelphia 19107, USA

The file includes Supplementary Fig. 1-11 and Supplementary Table 1-2

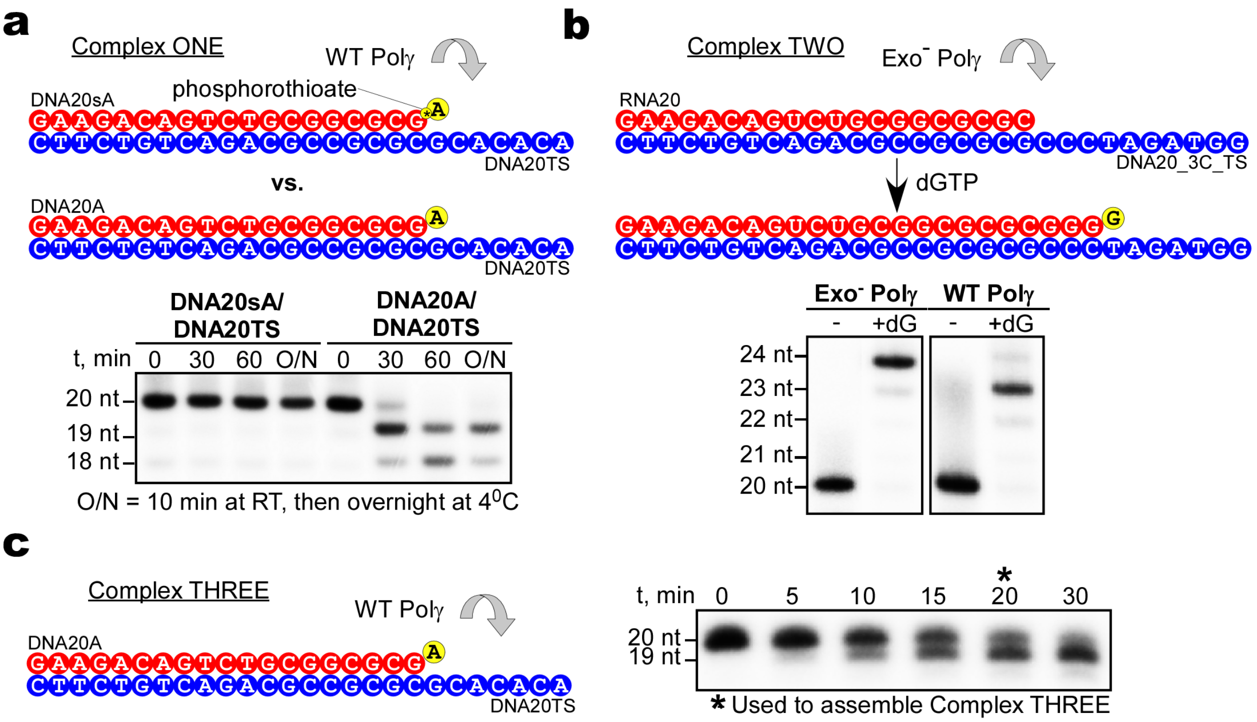

Supplementary Fig. 1: Assembly and activity of the Polγ complexes.

a. Topology of the DNA scaffolds with (DNA20sA/DNA20TS) and without (DNA20A/DNA20TS) a phosphorothioate bond connecting the terminal mismatched base (top panel). Exonuclease assays were performed using Wild Type (WT) Polγ and the scaffolds above for the times indicated (bottom panel).

b. Primer extension and misincorporation by Exo- Polγ. Scaffold (top panel) allowed for incorporation of three dGMP residues and misincorporation of a dGMP residue, generating a G-T mismatch (left panel). Extension of the primer by 3 nt is observed by WT Polγ (right panel).

c. Exonuclease assays by Wild Type (WT) Polγ on the (DNA20A/DNA20TS) scaffold (left panel) for the times indicated (right panel) for Complex THREE.

Gels in **a-c** are representative results from triplicate experiments.

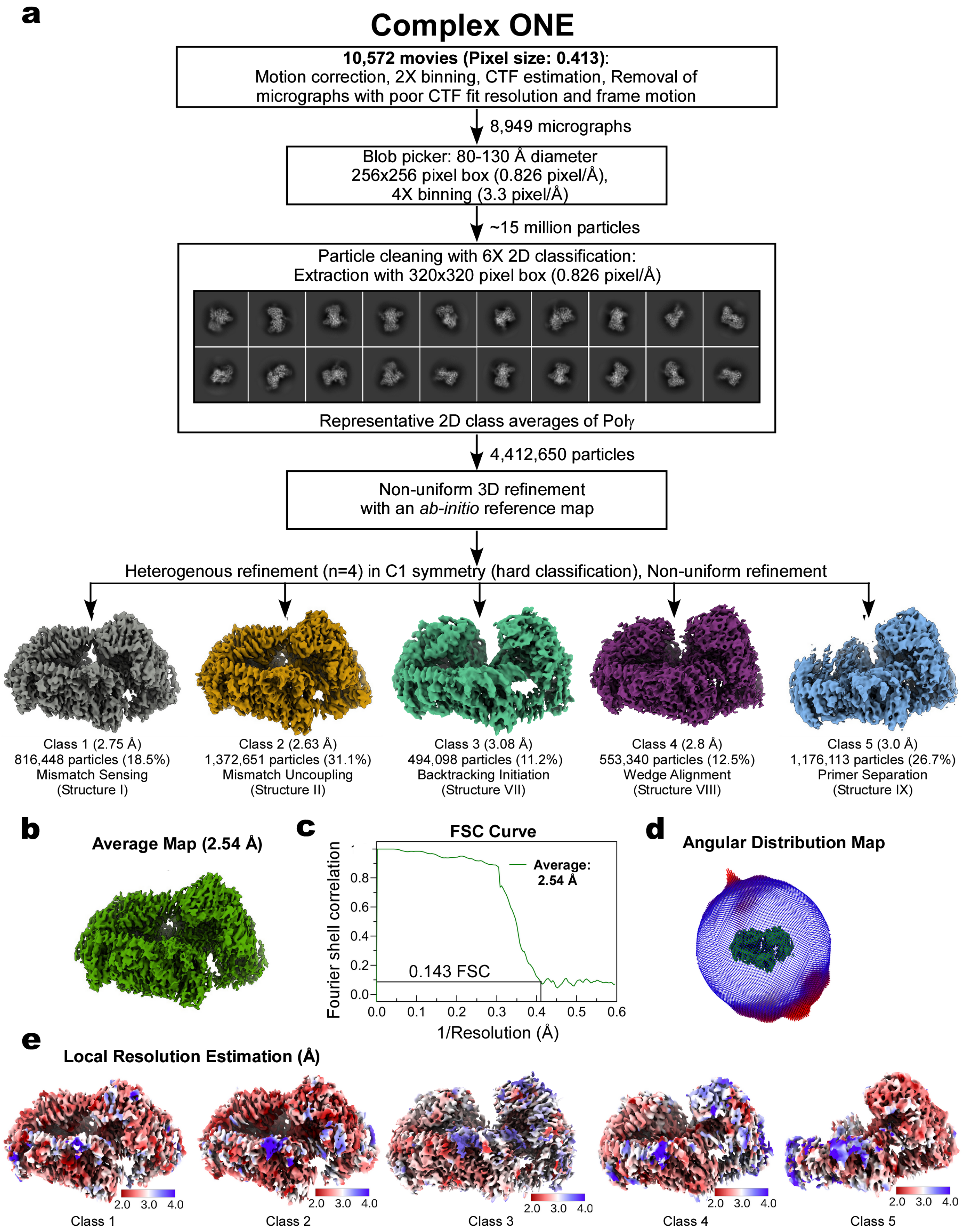
a
Complex ONE
10,572 movies (Pixel size: 0.413):
Motion correction, 2X binning, CTF estimation, Removal of micrographs with poor CTF fit resolution and frame motion
8,949 micrographs
Blob picker: 80-130 Å diameter
256x256 pixel box (0.826 pixel/Å),
4X binning (3.3 pixel/Å)
~15 million particles
Particle cleaning with 6X 2D classification:
Extraction with 320x320 pixel box (0.826 pixel/Å)
Representative 2D class averages of Polγ
4,412,650 particles
Non-uniform 3D refinement
with an ab-initio reference map
Heterogenous refinement (n=4) in C1 symmetry (hard classification), Non-uniform refinement
Class 1 (2.75 Å)
816,448 particles (18.5%)
Mismatch Sensing
(Structure I)
Class 2 (2.63 Å)
1,372,651 particles (31.1%)
Mismatch Uncoupling
(Structure II)
Class 3 (3.08 Å)
494,098 particles (11.2%)
Backtracking Initiation
(Structure VII)
Class 4 (2.8 Å)
553,340 particles (12.5%)
Wedge Alignment
(Structure VIII)
Class 5 (3.0 Å)
1,176,113 particles (26.7%)
Primer Separation
(Structure IX)
b
Average Map (2.54 Å)
c
FSC Curve
Average:
2.54 Å
Fourier shell correlation
0.143 FSC
1/Resolution (Å)
0.0
0.1
0.2
0.3
0.4
0.5
0.6
0.8
d
Angular Distribution Map
e
Local Resolution Estimation (Å)
2.0 3.0 4.0
Class 1
Class 2
Class 3
Class 4
Class 5

Supplementary Fig. 2: CryoEM image processing of Complex ONE.

a. Processing tree for Complex ONE

b. Average map of Polγ

c. Fourier shell correlation (FSC) plot for the average map

d. Angular distribution of Polγ particles contributing to the average map

e. Local resolution estimates for all 3D classes

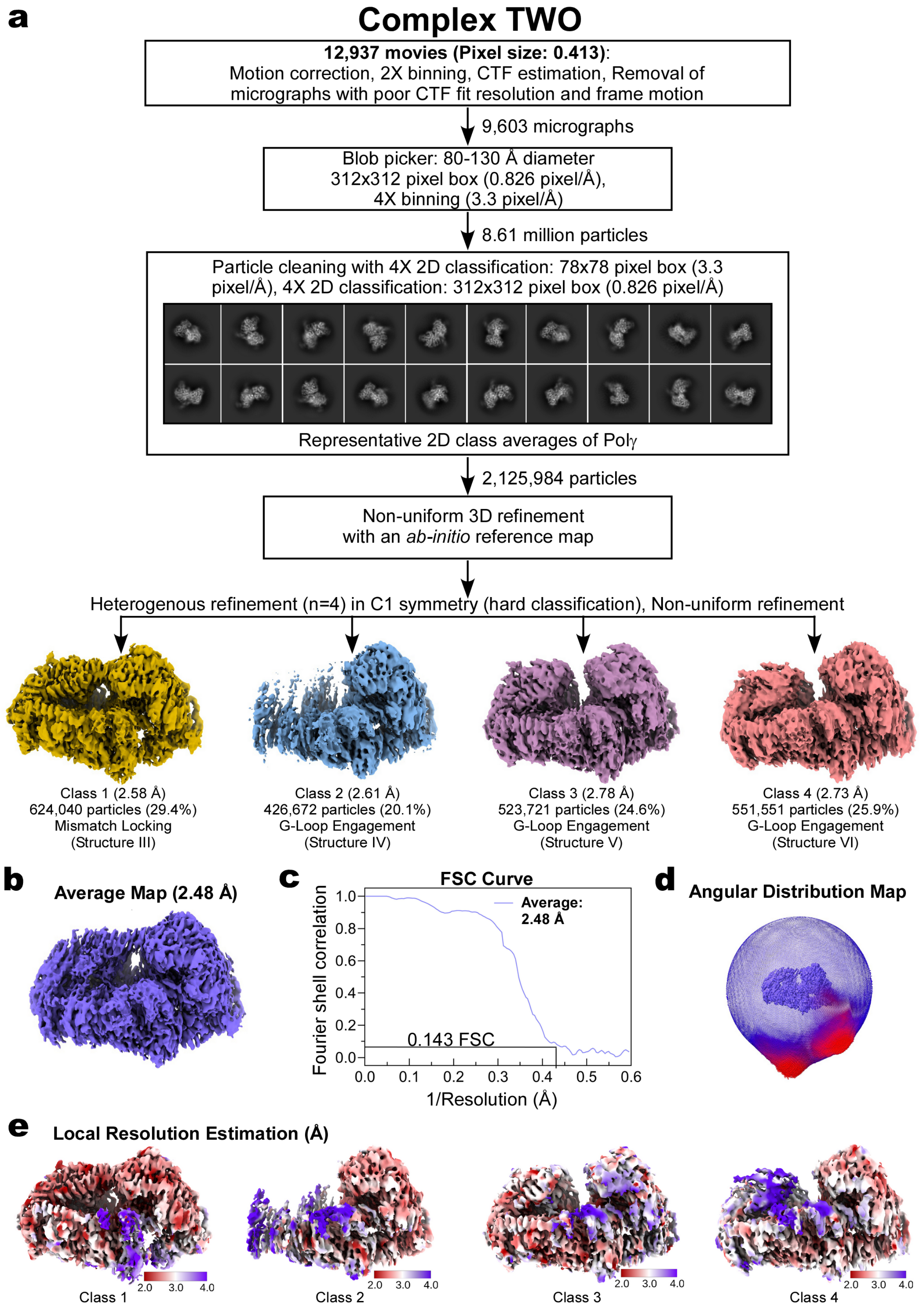
a
Complex TWO
12,937 movies (Pixel size: 0.413):
Motion correction, 2X binning, CTF estimation, Removal of micrographs with poor CTF fit resolution and frame motion
9,603 micrographs
Blob picker: 80-130 Å diameter
312x312 pixel box (0.826 pixel/Å),
4X binning (3.3 pixel/Å)
8.61 million particles
Particle cleaning with 4X 2D classification: 78x78 pixel box (3.3 pixel/Å), 4X 2D classification: 312x312 pixel box (0.826 pixel/Å)
Representative 2D class averages of Polγ
2,125,984 particles
Non-uniform 3D refinement
with an ab-initio reference map
Heterogenous refinement (n=4) in C1 symmetry (hard classification), Non-uniform refinement
Class 1 (2.58 Å)
624,040 particles (29.4%)
Mismatch Locking
(Structure III)
Class 2 (2.61 Å)
426,672 particles (20.1%)
G-Loop Engagement
(Structure IV)
Class 3 (2.78 Å)
523,721 particles (24.6%)
G-Loop Engagement
(Structure V)
Class 4 (2.73 Å)
551,551 particles (25.9%)
G-Loop Engagement
(Structure VI)
b
Average Map (2.48 Å)
c
FSC Curve
Average:
2.48 Å
Fourier shell correlation
0.143 FSC
1/Resolution (Å)
0.0
0.2
0.4
0.6
0.8
1.0
0.1
0.3
0.5
0.6
d
Angular Distribution Map
e
Local Resolution Estimation (Å)
2.0
3.0
4.0
Class 1
Class 2
Class 3
Class 4

Supplementary Fig. 3: CryoEM image processing of Complex TWO.

a. Processing tree for Complex TWO

b. Average map of Polγ

c. Fourier shell correlation (FSC) plot for the average map

d. Angular distribution of Polγ particles contributing to the average map

e. Local resolution estimates for all 3D classes

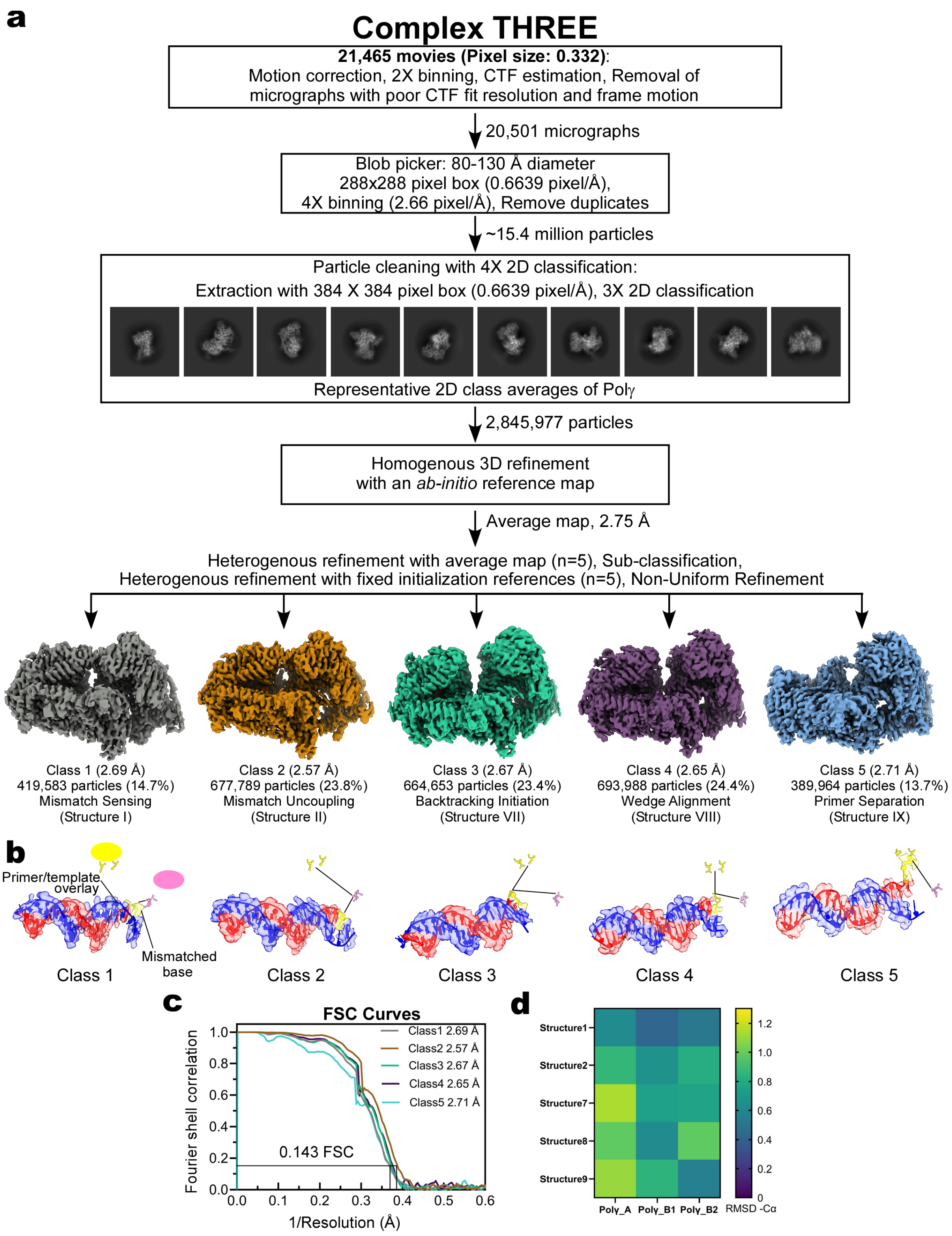
a
Complex THREE
21,465 movies (Pixel size: 0.332):
Motion correction, 2X binning, CTF estimation, Removal of micrographs with poor CTF fit resolution and frame motion
20,501 micrographs
Blob picker: 80-130 Å diameter
288x288 pixel box (0.6639 pixel/Å),
4X binning (2.66 pixel/Å), Remove duplicates
~15.4 million particles
Particle cleaning with 4X 2D classification:
Extraction with 384 X 384 pixel box (0.6639 pixel/Å), 3X 2D classification
Representative 2D class averages of Polγ
2,845,977 particles
Homogenous 3D refinement
with an ab-initio reference map
Average map, 2.75 Å
Heterogenous refinement with average map (n=5), Sub-classification,
Heterogenous refinement with fixed initialization references (n=5), Non-Uniform Refinement
Class 1 (2.69 Å)
419,583 particles (14.7%)
Mismatch Sensing
(Structure I)
Class 2 (2.57 Å)
677,789 particles (23.8%)
Mismatch Uncoupling
(Structure II)
Class 3 (2.67 Å)
664,653 particles (23.4%)
Backtracking Initiation
(Structure VII)
Class 4 (2.65 Å)
693,988 particles (24.4%)
Wedge Alignment
(Structure VIII)
Class 5 (2.71 Å)
389,964 particles (13.7%)
Primer Separation
(Structure IX)
b
Primer/template overlay
Mismatched base
Class 1
Class 2
Class 3
Class 4
Class 5
c
FSC Curves
Fourier shell correlation
1/Resolution (Å)
0.143 FSC
Class1 2.69 Å
Class2 2.57 Å
Class3 2.67 Å
Class4 2.65 Å
Class5 2.71 Å
d
Structure1
Structure2
Structure7
Structure8
Structure9
Poly_A
Poly_B1
Poly_B2
RMSD -Cα

Supplementary Fig. 4: CryoEM image processing of Complex THREE.

a. Processing tree for Complex THREE

b. CryoEM density maps for DNA in Complex THREE overlayed with their corresponding fitted models from Complex ONE

c. Fourier shell correlation (FSC) plots for all classes

d. Superimposition analysis of refined Polγ structures of Complex THREE and Complex ONE

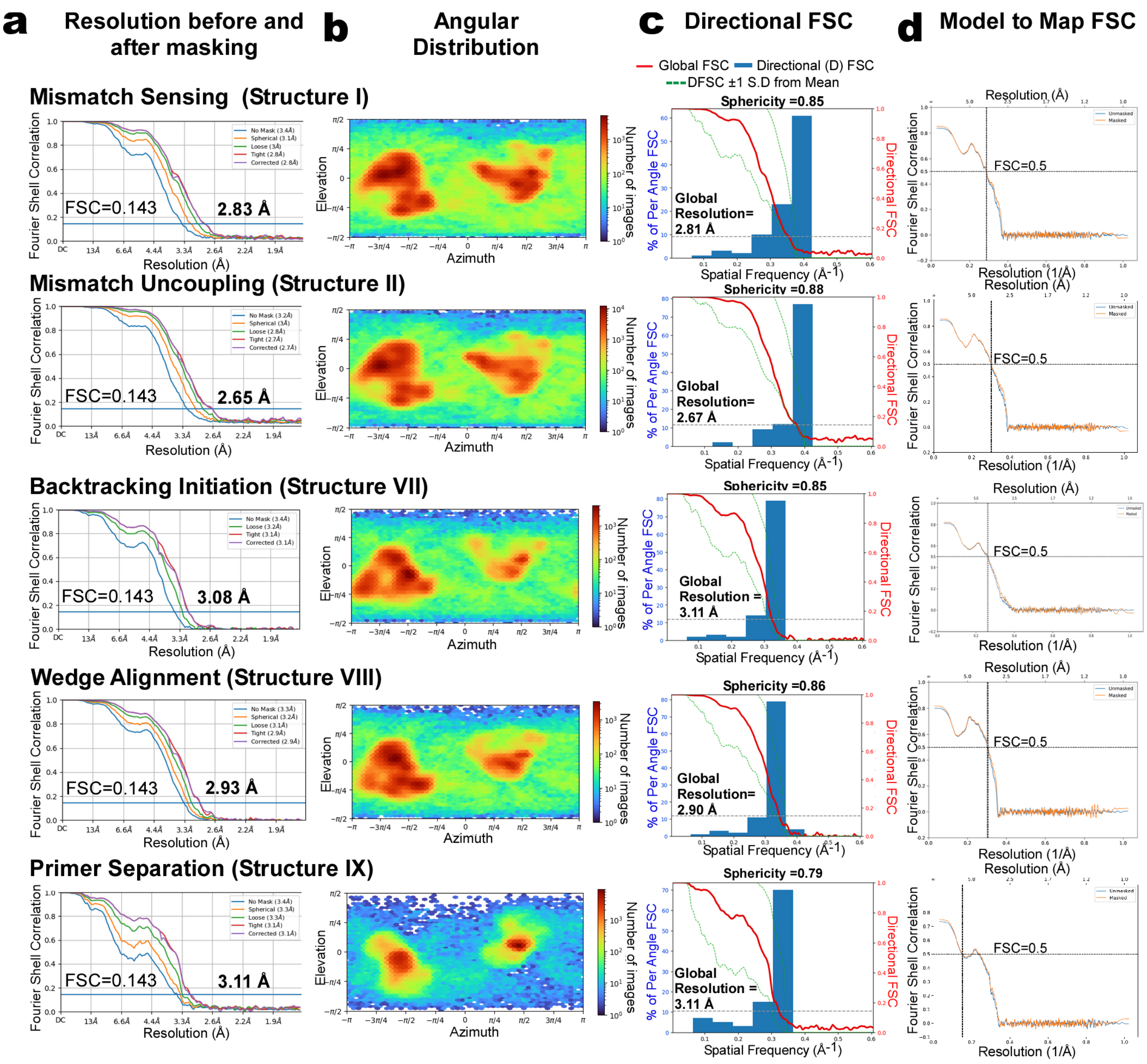

Supplementary Fig. 5: CryoEM data quality for Complex ONE.

a-d. Resolution estimates (**a**), angular distribution (**b**), directional FSC plots (**c**), and model correlation (**d**) of CryoEM reconstructions of the Polγ complexes. The vertical line in **d** represents resolution at FSC=0.5.

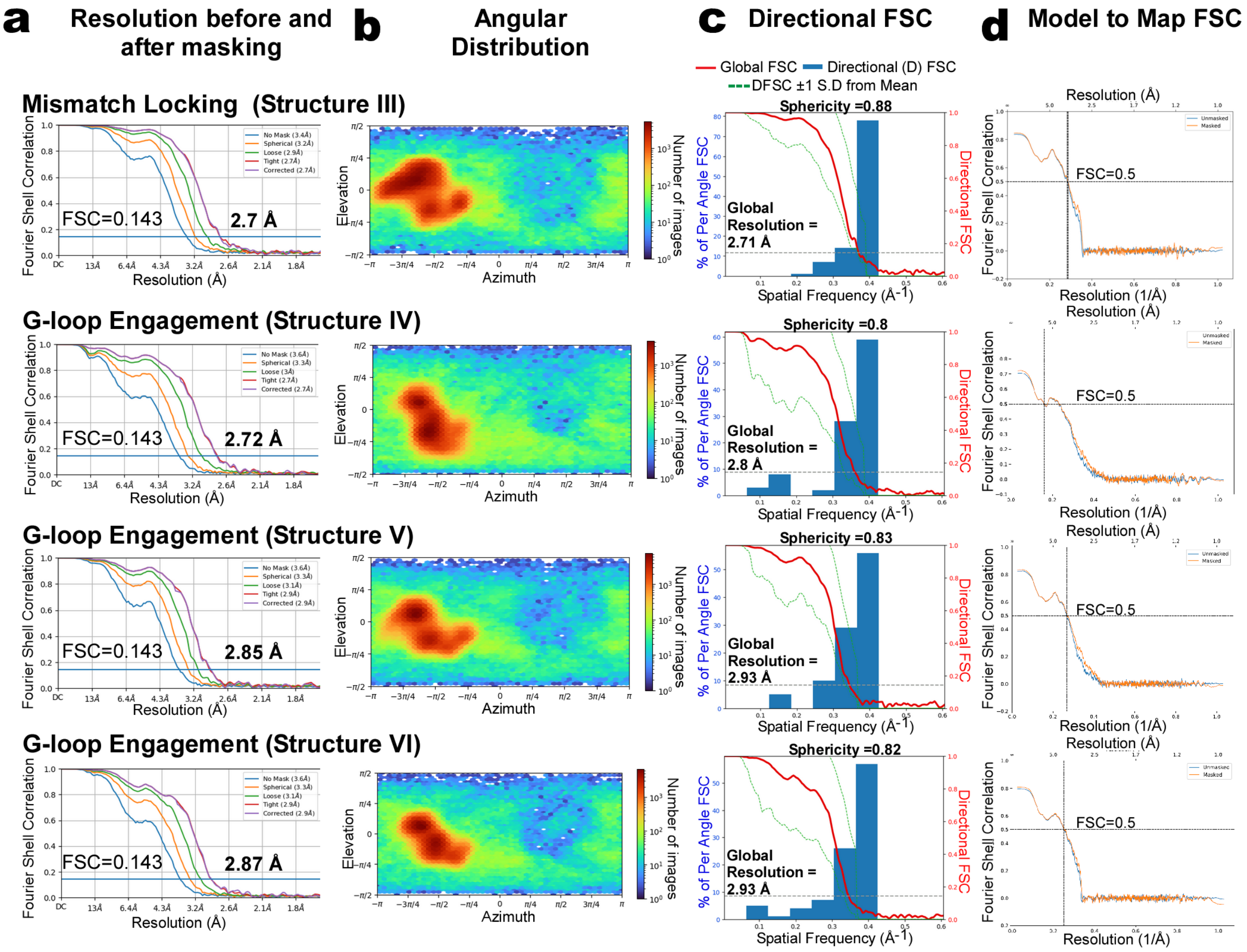

Supplementary Fig. 6: CryoEM data quality for Complex TWO.

a-d. Resolution estimates (**a**), angular distribution (**b**), directional FSC plots (**c**), and model correlation (**d**) of CryoEM reconstructions of the Polγ complexes. The vertical line in **d** represents resolution at FSC=0.5.

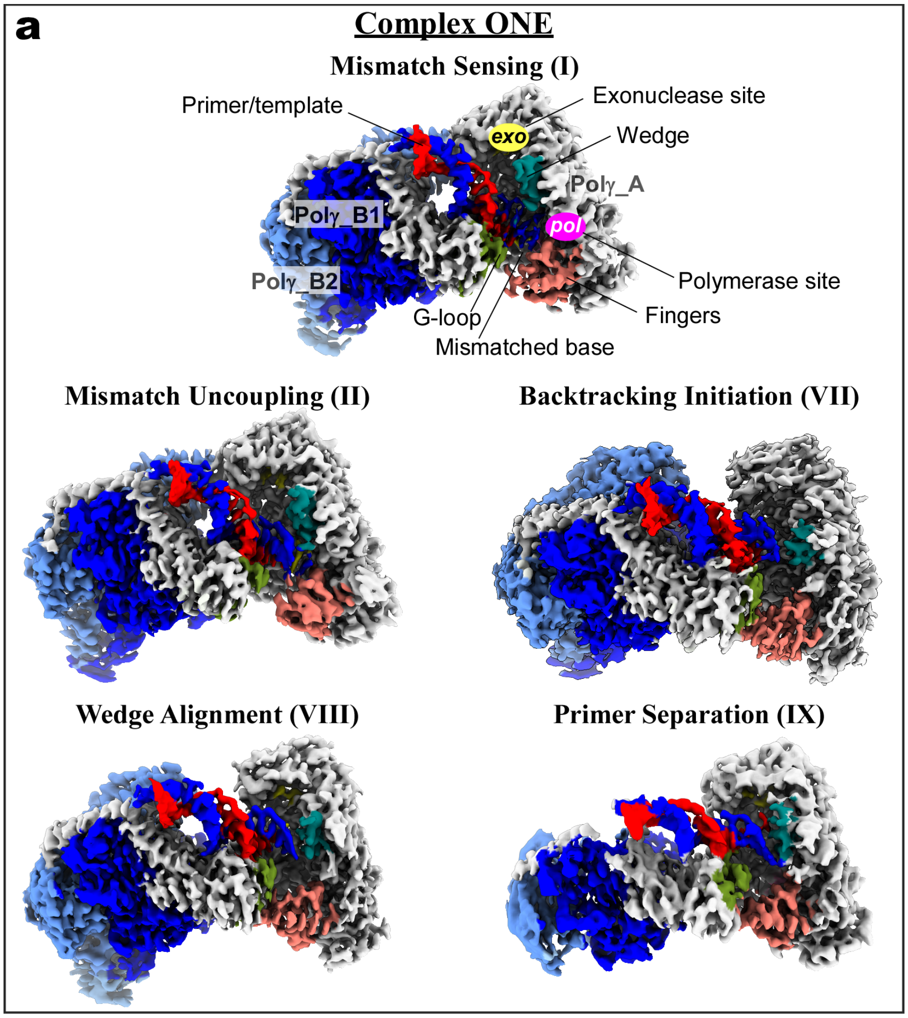

a
Complex ONE
Mismatch Sensing (I)
Primer/template
Exonuclease site
exo
Wedge
Polγ_A
Polγ_B1
pol
Polγ_B2
Polymerase site
Fingers
G-loop
Mismatched base
Mismatch Uncoupling (II)
Backtracking Initiation (VII)
Wedge Alignment (VIII)
Primer Separation (IX)

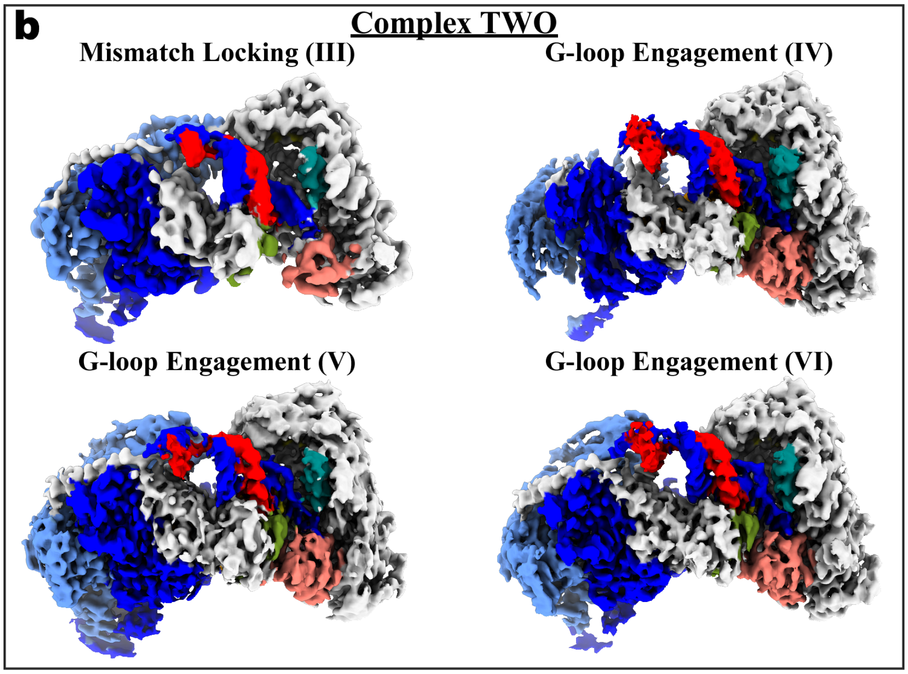

b
Complex TWO
Mismatch Locking (III)
G-loop Engagement (IV)
G-loop Engagement (V)
G-loop Engagement (VI)

Supplementary Fig. 7: CryoEM density maps for the proofreading complexes.

a. Complex ONE (Structures I, II, VII, VIII, IX)

b. Complex TWO (Structures III, IV, V, and VI)

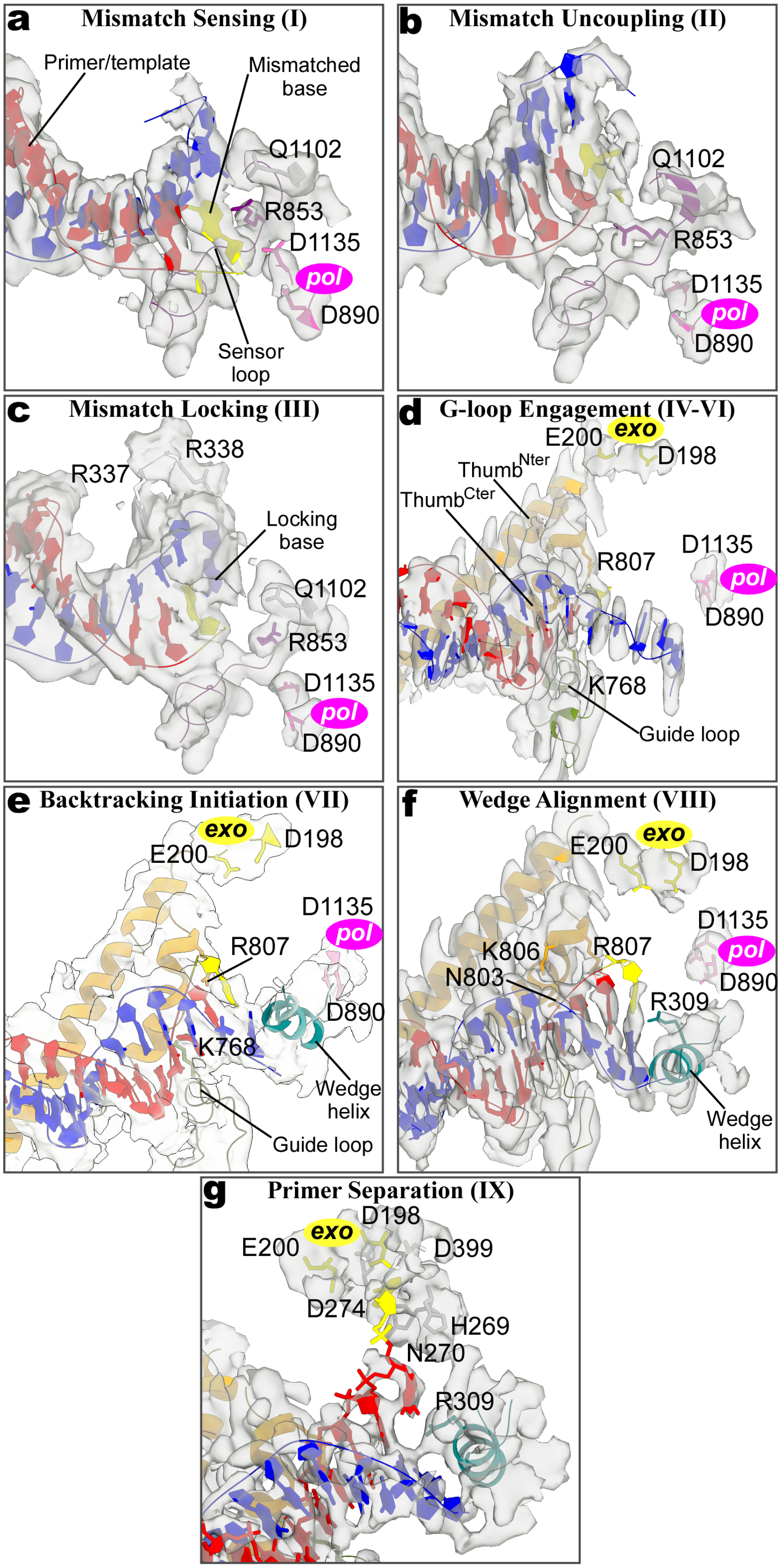
a
Mismatch Sensing (I)
Primer/template
Mismatched base
Q1102
R853
D1135
pol
D890
Sensor loop
b
Mismatch Uncoupling (II)
Q1102
R853
D1135
pol
D890
c
Mismatch Locking (III)
R338
R337
Locking base
Q1102
R853
D1135
pol
D890
d
G-loop Engagement (IV-VI)
E200
exo
D198
Thumb^Nter
Thumb^Cter
R807
D1135
pol
D890
K768
Guide loop
e
Backtracking Initiation (VII)
exo
D198
E200
D1135
pol
R807
D890
K768
Wedge helix
Guide loop
f
Wedge Alignment (VIII)
E200
exo
D198
D1135
K806
R807
pol
N803
D890
R309
Wedge helix
g
Primer Separation (IX)
D198
E200
exo
D399
D274
H269
N270
R309

Supplementary Fig. 8: Representative Coulombic densities and model fits of the key structural elements involved in proofreading.

a. Mismatched base in the *pol* active site of Polγ in the Mismatch Sensing complex.

b. Primer-template DNA in the Mismatch Uncoupling complex.

c. Primer-template DNA in the Mismatch Locking complex.

d. Primer-template DNA in the Guide Loop Engagement complex.

e. Primer-template DNA in the Backtracking Initiation complex

f. Primer-template DNA in the Wedge complex.

g. Single-stranded DNA in the *exo* site of Polγ in Primer Separation complex. Local densities of the CryoEM maps (grey) are shown around 3 Å from the model.

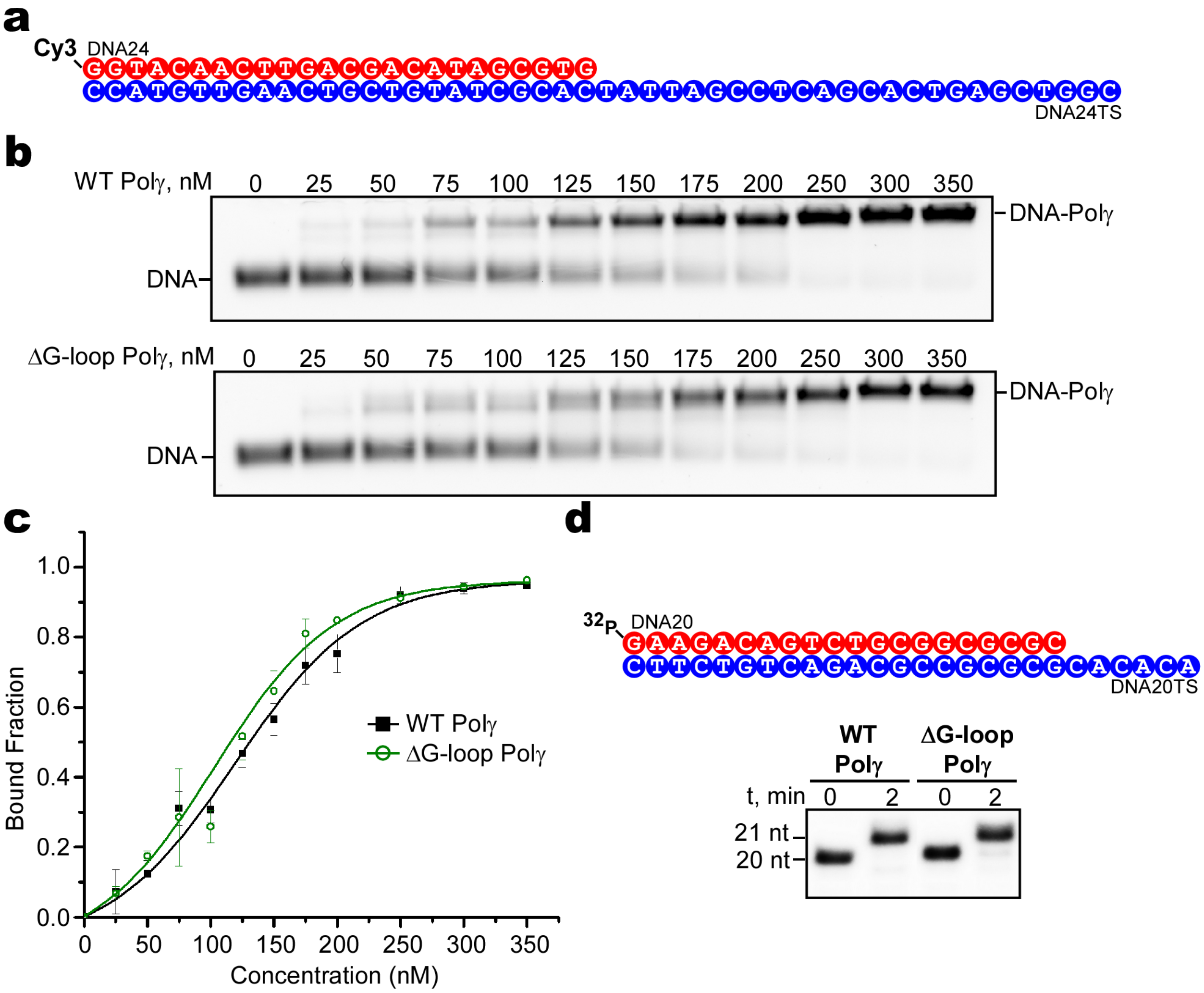

Supplementary Fig. 9: Deletion of the G-loop does not affect Polγ binding affinity.

a. Topology of the DNA scaffold (DNA24/DNA24TS) containing the 5'-Cy3 fluorescent label, used as a substrate for EMSA experiments.

b. EMSA using WT Polγ (top panel) and ΔG-loop Polγ (bottom panel).

c. Relative affinity of WT and ΔG-loop Polγ to the substrate (**a**) as observed in (**b**) (n=3 independent experiments). Error bars show standard deviation.

d. ΔG-loop Polγ is catalytically active. Primer extension by a single nucleotide, dGTP, was performed on the scaffold shown.

Gels in **b,d** are representative results from triplicate experiments.

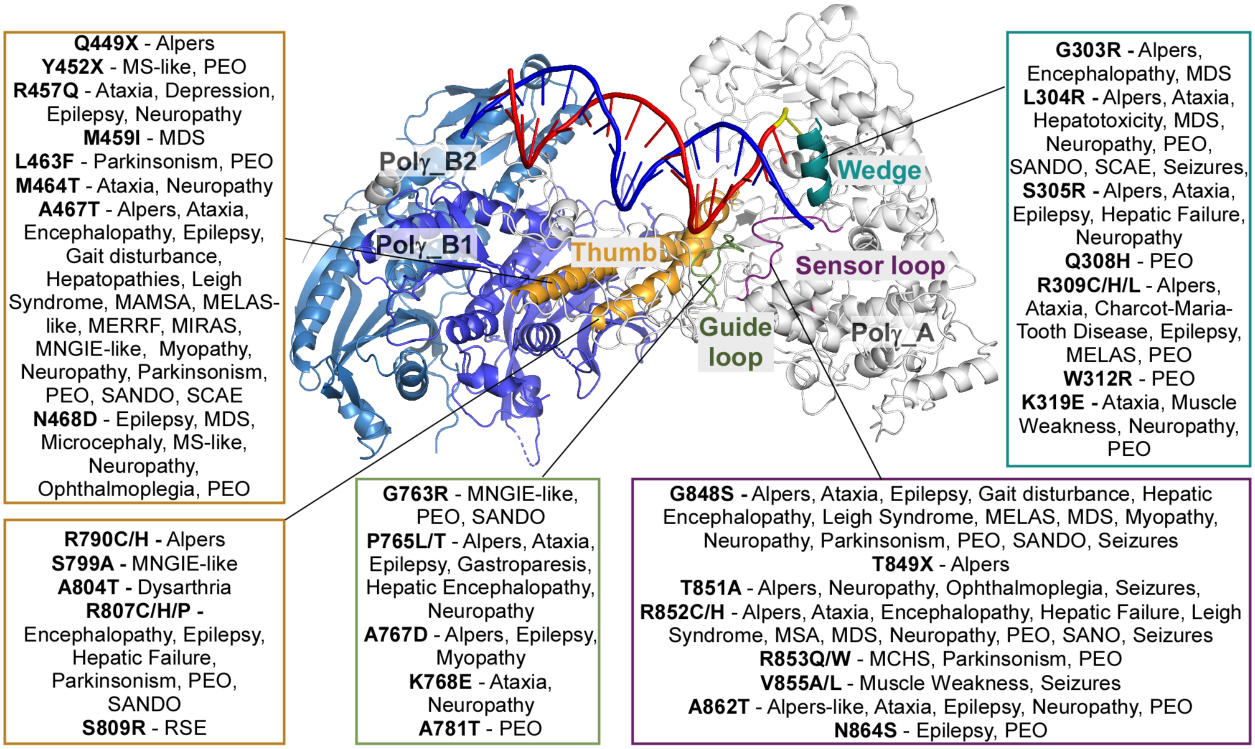

Supplementary Fig. 10: Residues in structural elements implicated in proofreading are associated with pathogenesis. Polγ Primer Separation complex (ribbon representation) is shown. The following databases were used as search tools: https://tools.niehs.nih.gov/polg/ and https://www.mitomap.org/polg/

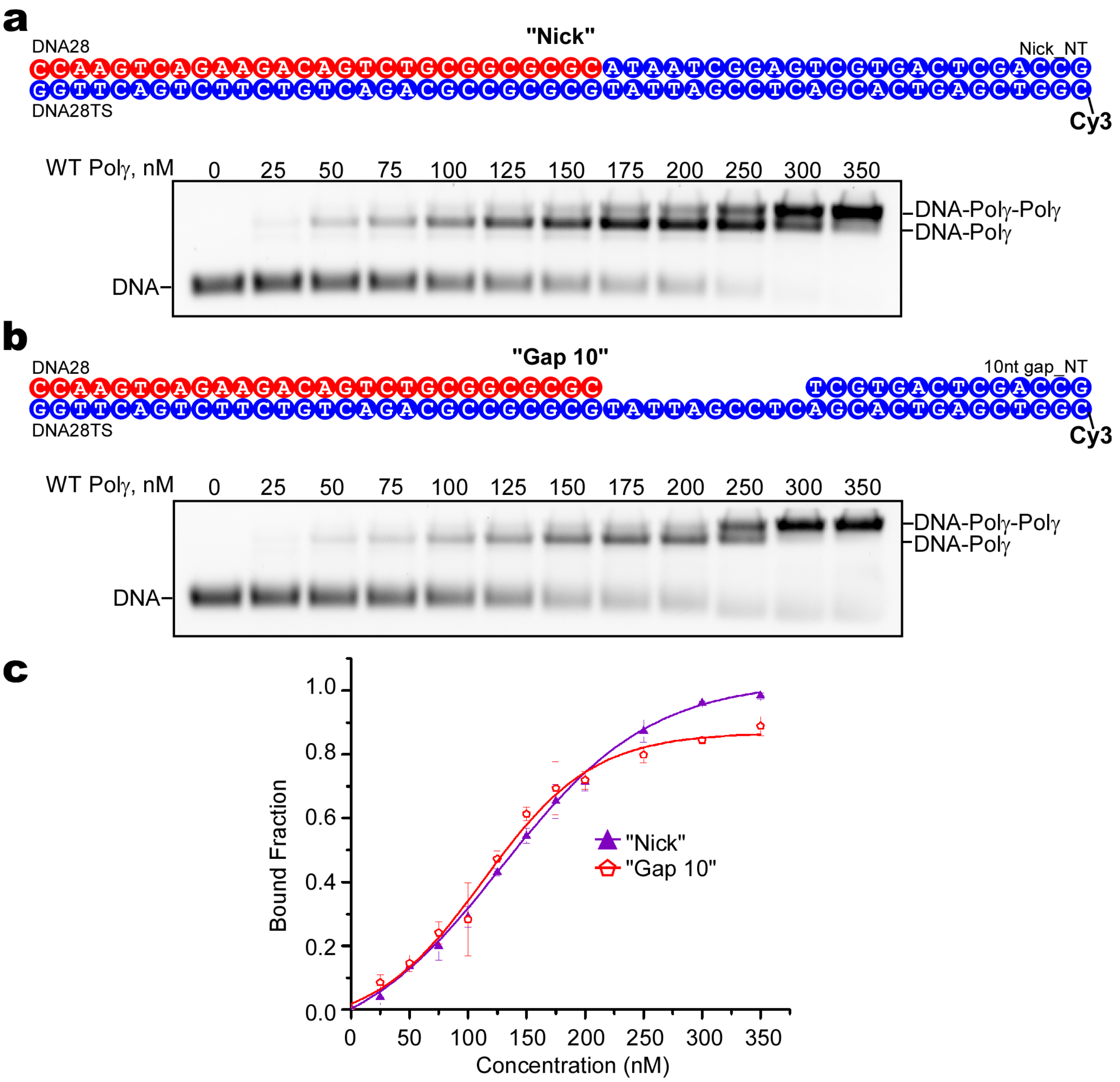

Supplementary Fig. 11: The presence of a nick does not affect the binding affinity of WT Polγ.

a,b. EMSA using WT Polγ on "Nick" (**a**) and "Gap 10" (**b**) substrates.

c. Relative affinity of WT Polγ to "Nick" (**a**) and "Gap 10" (**b**) substrates as observed in the EMSA experiments (n=3 independent experiments). Error bars show standard deviation.

Gels in **a**,**b** are representative results from triplicate experiments.

Supplementary Table 1: Summary of CryoEM data collection and image processing

		**Complex ONE**				**Complex TWO**			
**Data Acquisition**									
Electron Microscope		Titan Krios							
Voltage (kV)		300							
Detector		Gatan K3-Summit							
Magnification		105000							
Camera mode		Super-resolution							
Energy filter slit width (eV)		20							
Grid type		300 mesh Holey gold Ultra-AuFoil 1.2/1.3							
Software		SerialEM							
Pixel Size (Å)		0.4130				0.4128			
Focus range (µm)		-0.5 to -1.8				-0.8 to -2.0			
Total electron dose ($e^-/Å^2$)		70				60			
Dose rate (e^-/s/phys. Pixel)		19.35				15.92			
Number of frames		70				60			
Number of micrographs collected		10572				12937			
Number of micrographs used		8949				9603			
**Image Processing**	**Class 1**	**Class 2**	**Class 3**	**Class 4**	**Class 5**	**Class 1**	**Class 2**	**Class 3**	**Class 4**
Program		CryoSPARC 3.2							
Number of particles picked (million)		15				8.61			
Number of particles (After 2D classification)	4412650					2125984			
Number of particles (After 3D classification)	816448	1372651	494098	553340	1176113	624040	426672	523721	551551
Symmetry imposed		C1							
Map Resolution FSC 0.143 (Å)	2.75	2.63	3.08	2.8	3.0	2.58	2.61	2.78	2.73
Resolution, Polγ-A (Å)	2.87	2.54	3.18	2.93	2.75	2.74	2.74	2.99	2.86
Resolution, Polγ-B (Å)	2.75	2.61	2.94	2.81	-	2.65	-	2.79	2.92
EMDB ID	EMD-29745	EMD-29746	EMD-41091	EMD-29747	EMD-29748	EMD-29749	EMD-29751	EMD-29752	EMD-29750

Supplementary Table 2: Model composition, refinement, and validation statistics

	Complex ONE					Complex TWO			
**Composition**	**Class 1**	**Class 2**	**Class 3**	**Class 4**	**Class 5**	**Class 1**	**Class 2**	**Class 3**	**Class 4**
Initial Model used (PDB code)	4ztz								
Nonhydrogen atoms	14084	14544	14271	13633	12623	14673	11952	14500	14485
Protein residues	1671	1739	1706	1637	1634	1747	1716	1734	1734
Nucleotides	37	39	36	36	39	46	38	38	38
**Refinement**									
RMS Deviation (Bonds), Å	0.004	0.006	0.005	0.009	0.009	0.006	0.005	0.006	0.006
RMS Deviation (Angle), °	0.79	1.03	1.08	1.08	1.04	1.04	0.82	1.04	1.00
Model to map CC (mask)*	0.71 (0.70)	0.69 (0.74)	0.62 (0.61)	0.70 (0.67)	0.55 (0.66)	0.69 (0.74)	0.53 (0.71)	0.65 (0.63)	0.61 (0.71)
**Ramachandran Plot**									
Preferred (%)	93.37	94.05	92.2	91.37	92.22	89.97	94.32	92.23	92.64
Allowed (%)	6.63	5.95	7.8	8.63	7.78	10.03	5.68	7.77	7.36
Outliers (%)	0.00	0.00	0.00	0.00	0.00	0.00	0.00	0.00	0.00
**Validation**									
Clash-score	7.57	6.21	6.78	7.47	6.95	7.89	5.67	7.89	6.65
MolProbity score	1.94	2.00	1.85	2.18	2.12	2.07	1.87	2.19	2.14
Poor rotamers (%)	1.33	2.2	0.69	2.2	2.17	1.31	1.72	2.33	1.98
PDB ID	8G5I	8G5J	8T7E	8G5K	8G5L	8G5M	8G50	8G5P	8G5N

*CCmax Polγ (Polγ_A)
