# Supplementary material for: Structural basis for DNA proofreading
Source: Nat Commun. 2023 Dec 27;14:8501. doi: 10.1038/s41467-023-44198-8 (PMC10752894; doi:10.1038/s41467-023-44198-8)
Supplement: Supplementary file 3 — Description of Additional Supplementary files [file 41467_2023_44198_MOESM3_ESM.docx]

**Description of Additional Supplementary Files:**

File Name: Supplementary Video 1

Description: A close-up view of the DNA proofreading process

File Name: Supplementary Video 2

Description: Proofreading by DNA polymerase Gamma

File Name: Supplementary Video 3

Description: Transition from Mismatch Sensing to Mismatch Uncoupling

File Name: Supplementary Video 4

Description: Transition from Mismatch Uncoupling to Mismatch Locking

File Name: Supplementary Video 5

Description: Transition from Mismatch Locking to Guide Loop Engagement

File Name: Supplementary Video 6

Description: Transition from Wedge Alignment to Primer Separation
